# Cerebral hemodynamic changes to transcranial Doppler sonography in celiac disease: A pilot study

**DOI:** 10.3389/fnhum.2022.931727

**Published:** 2022-09-06

**Authors:** Francesco Fisicaro, Giuseppe Lanza, Carmela Cinzia D’Agate, Manuela Pennisi, Mariagiovanna Cantone, Giovanni Pennisi, Marios Hadjivassiliou, Rita Bella

**Affiliations:** ^1^Department of Biomedical and Biotechnological Sciences, University of Catania, Catania, Italy; ^2^Department of Surgery and Medical-Surgical Specialties, University of Catania, Catania, Italy; ^3^Clinical Neurophysiology Research Unit, Oasi Research Institute-IRCCS, Troina, Italy; ^4^Gastroenterology and Endoscopy Unit, Policlinico University Hospital “G. Rodolico-San Marco”, Catania, Italy; ^5^Neurology Unit, Policlinico University Hospital “G. Rodolico-San Marco”, Catania, Italy; ^6^Department of Neurology, Sant’Elia Hospital, ASP Caltanissetta, Caltanissetta, Italy; ^7^Academic Department of Neurosciences, Sheffield Teaching Hospitals NHS Foundation Trust, Royal Hallamshire Hospital, Sheffield, United Kingdom; ^8^Department of Medical and Surgical Sciences and Advanced Technologies, University of Catania, Catania, Italy

**Keywords:** celiac disease, cerebral blood flow, cerebral hemodynamics, vasomotor reactivity, transcranial Doppler sonography, cognition, depression

## Abstract

**Background:**

Sonographic mesenteric pattern in celiac disease (CD) suggests a hyperdynamic circulation. Despite the well-known CD-related neurological involvement, no study has systematically explored the cerebral hemodynamics to transcranial Doppler sonography.

**Materials and methods:**

Montreal Cognitive Assessment (MoCA) and 17-item Hamilton Depression Rating Scale (HDRS) were assessed in 15 newly diagnosed subjects with CD and 15 age-, sex-, and education-matched healthy controls. Cerebral blood flow (CBF) velocities and indices of resistivity (RI) and pulsatility (PI) from the middle cerebral artery (MCA), bilaterally, and the basilar artery (BA) were recorded. We also assessed cerebral vasomotor reactivity (CVR) through the breath-holding test (BHT).

**Results:**

Worse scores of MoCA and HDRS were found in patients compared to controls. Although patients showed higher values of CBF velocity from MCA bilaterally compared to controls, both at rest and after BHT, no comparison reached a statistical significance, whereas after BHT both RI and PI from BA were significantly higher in patients. A significant negative correlation between both indices from BA and MoCA score were also noted.

**Conclusion:**

These treatment-naïve CD patients may show some subtle CVR changes in posterior circulation, thus possibly expanding the spectrum of pathomechanisms underlying neuroceliac disease and in particular gluten ataxia. Subclinical identification of cerebrovascular pathology in CD may help adequate prevention and early management of neurological involvement.

## Introduction

It is known that, within the wide spectrum of gluten-related disorders (Hadjivassiliou et al., 2014), the typical celiac disease (CD) is the so-called “tip of the iceberg” (Hopper et al., 2007), given that, for each individual affected, 5-to-6 patients show atypical manifestations (Green et al., 2005). Therefore, CD should be now considered as a multiorgan disorder with different clinical features.

Regarding extra-intestinal symptoms, neuropsychiatric and neurological manifestations still represent a challenge for diagnosis, since they can co-occur or precede CD, or even exist in isolation without any gastrointestinal symptoms (Hadjivassiliou et al., 2002b,^2010^, ^2014^; Briani et al., 2008). In the recent prospective investigation of newly diagnosed CD subjects (Hadjivassiliou et al., 2019), neurological involvement was frequent and a relevant decreased volume of some brain areas was noted in patients with tissue transglutaminase (tTG)-6 antibodies. Moreover, at neurological consultation, most of patients with confirmed CD already showed alterations at the magnetic resonance imaging (MRI) of the brain (Currie et al., 2012). As such, a technique allowing early diagnosis, reliable follow-up, and careful management of CD is desirable.

Transcranial Doppler sonography (TCD) represents a non-invasive ultrasound method using a ≤2 MHz (low-frequency) probe transducer to insonate cerebral basal arteries through some specifically thin skull windows. TCD is used for an *in vivo* assessment of cerebral blood flow (CBF) vessel velocity and resistance on a prolonged period and with high temporal resolution (Sharma et al., 2016; Hakimi et al., 2020). Most of the evidence supports the prognostic and preventive role of TCD in patients with sickle cell disorder (Adams et al., 1997), subarachnoid hemorrhage (Lysakowski et al., 2001), stroke (Demchuk et al., 2000), monitoring of the thrombolysis in brain ischemia (Alexandrov and Grotta, 2002), intensive care neuromonitoring (Vinciguerra and Bösel, 2017), right-to-left cardiopulmonary shunt, and brainstem death diagnosis (Ringelstein et al., 1998). Recently, our research group has applied TCD to evaluate the presence and severity of cerebral small vessel pathology and cognitive impairment (mostly executive dysfunction) in older subjects at risk for stroke (Vagli et al., 2020) or dementia (Puglisi et al., 2018; Vinciguerra et al., 2019).

Transcranial Doppler sonography has been applied also to probe cerebral autoregulation mechanisms (Panerai, 1998): integrated with morphology of the waveform, specific indexed calculated from flow velocities, including the Pourcelot’s resistivity index (RI) and the Gosling’s pulsatility index (PI), allow the assessment of a hyperdynamic flow status, vasospasm, and increased vascular resistances. In addition, cerebral vasomotor reactivity (CVR) to TCD may be non-invasively and accurately evaluated by means of the vasoconstrictive or vasodilatory ability of the brain resistance arterioles (Ringelstein et al., 1988; Aaslid et al., 1989; Ficzere et al., 1997). Modulating the systemic blood pressure (and, subsequently, the perfusion pressure of the brain) allows for probing the cerebral autoregulatory capacity, while different types of stimulus (e.g., the acetazolamide intake, the CO_2_ inhalation) determines a hemodynamic reaction which correlates with the brain metabolic response secondary to the stimulus itself (Ringelstein et al., 1988; Widder, 1992; Dahl et al., 1994). In recent years, more physiological stimuli have been adopted, including the breath-holding test (BHT) and the voluntary hyperventilation (Settakis et al., 2002), both of which are fully tolerated and recommended for testing the CVR in subjects with stable pulmonary disease or conditions impairing the brain microvasculature (Ficzere et al., 1997; Fülesdi et al., 1997).

In CD patients, the sonographic pattern of mesenteric circulation has been investigated by different studies, which demonstrate a hyperdynamic state, that reverts to normal after a gluten-free diet (GFD) (Fraquelli et al., 2006). Splanchnic blood flow to Doppler ultrasound, i.e., in the superior mesenteric artery and portal vein, has been assessed in CD subjects both in basal and post-prandial conditions. In fasting condition, there was an increased flow and velocity in both the superior mesenteric artery and portal vein compared to healthy individuals (Arienti et al., 1996; Aliotta et al., 1997; Giovagnorio et al., 1998; Magalotti et al., 2003). Conversely, in post-prandial phase, the above-mentioned indexes showed a remarkably lower variation with respect to normal controls (Aliotta et al., 1997; Magalotti et al., 2003). Of note, an adherent long-term GFD was able to revert these abnormalities (Fraquelli et al., 2006).

To date, however, no study has systematically explored cerebral hemodynamics in CD. We evaluated TCD in newly diagnosed CD patients with respect to normal individuals. The hypothesis was that TCD might identify even preclinical CVR or CBF differences in CD compared to controls.

## Materials and methods

### Study subjects

A group of 15 treatment-naïve consecutive patients [2 men; years, mean age ± standard deviation (SD): 34.07 ± 12.03], with the diagnosis of CD based on the European Society for Pediatric Gastroenterology Hepatology and Nutrition guidelines (Husby et al., 2020), were recruited from the Celiac Disease Regional Center, “G. Rodolico-San Marco” Policlinico University Hospital of Catania (Italy). A sample of 15 healthy subjects (2 men; years 33.80 ± 9.29) was age-matched as normal controls. Each participant was right-handed and on free diet when enrolled. Patients’ disease duration at the time of their diagnosis was 3.64 ± 1.78 years. Every participant was preliminarily screened for cardio-cerebrovascular risk factors by means of medical records, had normal extracranial vessel Doppler ultrasound, and did not take any medication, with the exception of a patient on L-thyroxine, but levels of thyroid hormones within normal limits.

We excluded the following subjects: age <18 years; any neurological (i.e., cerebrovascular diseases, movement disorders, major neurocognitive disorders, traumatic brain injury, epilepsy, demyelinating diseases, etc.) or psychiatric disorders (e.g., mood, bipolar, psychotic, obsessive–compulsive disorders); acute or chronic not compensated medical illnesses (i.e., kidney or liver failure, heart failure, coronary heart disease, etc.), as well as any condition causing a hyperdynamic state (such as pregnancy, fever, anemia, thyrotoxicosis, liver cirrhosis); alcohol dependency or illicit drug intake; use of any drug affecting CBF or CVR; bilateral insufficient acoustic bone windows. Since iron-deficiency anemia may be a common presenting manifestation of CD, we have excluded subjects with a moderate-to-severe anemia, whereas those with mild anemia (i.e., hemoglobin level not lower than 10.0 g/dL), especially without low hematocrit values, were considered for possible inclusion.

Demographic and clinical evaluation included: sex, age, education, handedness, neurological and general examination, any comorbidity. A global test of the overall cognitive functioning through the Montreal Cognitive Assessment (MoCA), adjusted for education and age of every participant (Nasreddine et al., 2005), and a quantification of depressive symptoms by means of the 17-item Hamilton Depression Rating Scale (HDRS) (Hamilton, 1960) were carried out by one of the co-authors (F.F), who was blind to the subject’s allocation (i.e., control or patient). Additionally, computed tomography (CT) of the brain was performed in CD participants with a General Electric 64-slice helical scanner (slice thickness: 2.5 mm) to identify any intracranial calcification (which may be observed in CD) and to rule out overt neuroimaging alterations.

The Ethics Committee of “G. Rodolico-San Marco” Policlinico University Hospital of Catania (Italy) approved the study (Prot. n. 103/694). Written informed consent was obtained from the individuals for the publication of any potentially identifiable images or data included in this article. Every procedure was conducted according to the in Declaration of Helsinki (1964) and later amendments, in a fully equipped Lab and by using the same system and under the same experimental conditions.

### Transcranial Doppler sonography procedures

An expert sonographer (R.B), who was blind with respect to the participants’ status as control or patient, performed all the procedures through a dedicated TCD Compumedics DWL, Multi-Dop^®^ X digital (2016, Singen, Germany). CBF velocities of the proximal tract of the middle cerebral artery (MCA), bilaterally, and of the basilar artery (BA) were obtained through a 2 MHz pulsed-wave Doppler ultrasound handheld probe from the transtemporal and suboccipital bone windows, respectively. All recordings were obtained both at rest and at the depth providing the optimal signal (i.e., MCA: 50–60 mm; BA: 80–90 mm). The measures of interest were: mean blood flow velocity (MBFV), peak systolic velocity (PSV), end-diastolic velocity (EDV), RI [according to the formula: (PSV–EDV)/PSV)], and PI [which was equal to: (PSV–EDV)/MBFV)] (Gosling and King, 1974). These indices were recorded following a stable recording period of 30 s and for at least 10 cardiac cycles, as recommended (D’Andrea et al., 2016a).

Cerebral vasomotor reactivity to 30-s BHT was assessed for BA and MCA, bilaterally. The vasodilatory stimulation through the breath-holding and the CO_2_-induced hypercapnia is able indeed to identify an altered vasomotor reserve of the brain, and an impaired reaction can detect an increase in the risk of cerebrovascular diseases (Müller et al., 1995). Specifically, after a normal inspiration and without doing a Valsalva maneuver, participants had to hold the breath for 30 s; the breath-holding index was then obtained according to the following formula: [(PSV after breath holding–PSV at rest)/PSV at rest/time of breath holding] × 100. After a resting period of 2 min, the task was repeated and the mean of the two values was analyzed (Markus and Harrison, 1992). MCA-derived measures, both in resting state and during the CVR procedure, were calculated as a mean of two measurements on each side.

Transcranial Doppler sonography values on MCA and BA were recorded continuously both at rest and after the breath-holding period. All data collected were stored in the Lab PC for off-line analyses.

### Statistical analysis

All statistical analyses were performed by using the GraphPad Prism v.8. Data distribution was evaluated by means of the Shapiro-Wilk and Kolmogorov-Smirnov tests. Mann–Whitney test or Unpaired *t*-test were used to establish any statistical difference existing between CD patients and normal individuals for the anthropometric and biological variables measured. The Spearman’s rank correlation coefficient (*r*) was utilized to assess correlations existing between variables. *P*-values were statistically significant when <0.05.

## Results

Table 1 illustrates the relevant clinical data and diagnostic results. The Edinburgh Handedness Inventory confirmed the right-handedness of all participants (Oldfield, 1971). The general exam was unremarkable in all of them. Neurological examinations, except for isolated brisk tendon reflexes symmetrically at the upper limbs in a patient, was entirely normal. None of the participants had vascular risk factors; mean arterial pressure, heart rate, and oxygen saturation to pulse oximetry, recorded just before the TCD examination, were within the normal range in each participant. Comorbidities were identified in three patients: Raynaud phenomenon (one), autoimmune thyroiditis (one), and psoriasis and fibromyalgia (one). CT of the patients’ brain excluded intracranial calcifications, as well as any overt neuroimaging change. Table 2 shows that the groups were similar for sex, age, education, and anthropometric characteristics (weight, height, body mass index). MoCA (25.8 vs. 28.0, *p* = 0.004) and HDRS (8.3 vs. 2.9, *p* = 0.008) scored significantly worse in patients compared to controls.

**TABLE 1:** Relevant laboratory and clinical-instrumental data of subjects with celiac disease.

No	Age, years	Sex	Family history	Disease duration, years	Main clinical features	Comorbidity	Antibodies	Endoscopy	Histology
1	55	F	+	3.5	Mild iron-deficiency anemia, weight loss, dyspepsia, tiredness,	–	EMA, tTG	Duodenal folds scalloped	3c
2	18	F	+	1.5	Mild iron-deficiency anemia, asthenia	–	EMA, tTG	Duodenal folds scalloped	3c
3	25	F	+	5.5	Mild iron-deficiency anemia, dermatological manifestations, tiredness	–	EMA, tTG	Duodenal folds scalloped	3c
4	18	F	−	5.0	Mild iron-deficiency anemia, tiredness, headache, belly pain	–	EMA, tTG	Duodenal folds scalloped	3c
5	29	M	+	–	-(familial screening)	–	EMA, tTG	Duodenal folds scalloped	3c
6	45	M	−	3.5	Mild iron-deficiency anemia, weight loss, headache, abdominal pain, tiredness	–	tTG	Duodenal folds scalloped	3c
7	36	F	−	1.5	Mild iron-deficiency anemia, weight loss, vitamin D deficiency, headache, tiredness	Autoimmune thyroiditis	EMA, tTG	Duodenal folds scalloped	3c
8	27	F	−	6.0	Mild iron-deficiency anemia, weight loss, unsteadiness, tiredness, abdominal pain, diarrhea	–	EMA, tTG	Duodenal folds scalloped	3c
9	35	F	−	3.5	Mild iron-deficiency anemia, tiredness, nausea, abdominal pain, diarrhea	–	EMA, tTG	Duodenal folds scalloped	3c
10	44	F	+	6.0	Mild iron-deficiency anemia, headache, tiredness, stypsis and diarrhea	Fibromyalgia, psoriasis	tTG	Duodenal folds scalloped	3c
11	45	F	−	1.5	Tiredness, abdominal discomfort, diarrhea	Raynaud phenomenon	tTG	Villi moderately atrophic	3b
12	41	F	−	1.0	Mild iron-deficiency anemia, weight loss, tiredness, diffuse pain, dyspepsia, diarrhea	–	EMA, tTG	Duodenal folds scalloped	3c
13	49	F	−	5.5	Asthenia, tiredness, dyspepsia, alternate alvus	–	tTG	Duodenal folds scalloped	3c
14	24	F	−	4.0	Mild iron-deficiency anemia, weight loss, dyspepsia, tiredness	–	EMA, tTG	Duodenal folds scalloped	3c
15	20	F	−	3.0	Mild iron-deficiency anemia, tiredness,	–	EMA, tTG	Duodenal folds scalloped	3c

EMA, endomysial antibodies; F, female; M, male; tTG, tissue transglutaminase; histopathological classification based on the Marsh–Oberhuber grading system (Oberhuber, 2000): 3a, mildly flattening villi; 3b, severely flattening villi; 3c, totally flattening villi; +, positive/present; −, negative/absent.

**TABLE 2:** Demographic data and TMS features of the two groups of participants.

Variable	Healthy controls		CD patients		
	*Mean*	*SD*	*Mean*	*SD*	*p*
Height, m	1.68	0.09	1.62	0.08	0.083
Weight, Kg	60.07	8.19	57.87	17.38	0.104
BMI, Kg/m^2^	21.32	2.24	21.85	5.99	0.177
Education, years	15.87	4.44	14.60	3.44	0.058
MoCA	28.00	1.00	25.80	2.40	**0.004**
HDRS	2.87	2.20	8.27	6.30	**0.008**
R-MCA max,	90.87	13.92	101.4	23.22	0.143
R-MCA mean, cm/s	60.20	9.41	66.4	14.81	0.182
R-MCA min, cm/s	39.67	6.72	44.67	10.09	0.121
R-MCA PI	0.84	0.19	0.89	0.19	0.518
R-MCA RI	0.56	0.05	0.56	0.060	0.897
R-MCA-A max, cm/s	103.20	18.86	118.3	24.85	0.072
R- MCA-A mean, cm/s	75.73	15.69	84.73	17.58	0.150
R-MCA-A min, cm/s	50.40	13.39	59	15.04	0.109
R-MCA-A PI	0.67	0.10	0.68	0.09	0.894
R-MCA-A RI	0.49	0.05	0.49	0.04	0.882
R-MCA BHI	0.86	0.52	0.95	0.29	0.556
L-MCA max, cm/s	92.13	14.51	100.8	19.92	0.184
L-MCA mean, cm/s	59.67	10.19	67	14.77	0.125
L-MCA min, cm/s	39.47	7.03	45.13	9.501	0.074
L-MCA PI	0.85	0.18	0.89	0.15	0.462
L-MCA RI	0.56	0.05	0.57	0.06	0.654
L-MCA-A max, cm/s	104.1	15.83	118.1	25.94	0.084
L-MCA-A mean, cm/s	74.73	11.94	83.53	20.41	0.161
L-MCA-A min, cm/s	53.93	8.16	59.13	16.09	0.274
L-MCA-A PI	0.67	0.10	0.75	0.15	0.093
L-MCA-A RI	0.48	0.42	0.58	0.16	0.053
L-MCA BHI	0.87	0.40	0.83	0.55	0.616
BA max, cm/s	63.00	18.17	71.07	13.56	0.179
BA mean, cm/s	43.80	13.62	47.33	10.67	0.436
BA min, cm/s	28.13	10.11	31.8	8.00	0.280
BA PI	0.79	0.13	0.85	0.15	0.261
BA RI	0.55	0.06	0.59	0.06	0.110
BA-A max, cm/s	75.87	18.64	84.2	22.7	0.281
BA-A mean, cm/s	56.33	14.24	60.33	17.45	0.497
BA-A min, cm/s	38.33	10.42	40.93	14.61	0.579
BA-A PI	0.65	0.09	0.76	0.16	**0.023**
BA-A RI	0.48	0.06	0.53	0.08	**0.042**
BA BHI	1.03	0.44	0.94	0.98	0.091

A, value after apnea (i.e., breath-holding test); BA, basilar artery; BHI, breath-holding index; BMI, body mass index; CD, celiac disease; HDRS, 17-item Hamilton depression rating scale; L, left; max, peak blood flow systolic velocity; mean, mean blood flow velocity min = end-diastolic blood flow velocity; MoCA, Montreal cognitive assessment; PI, pulsatility index; R, right; RI, resistivity index; SD, standard deviation; bold numbers, statistically significant p-values.

**FIGURE 1:**
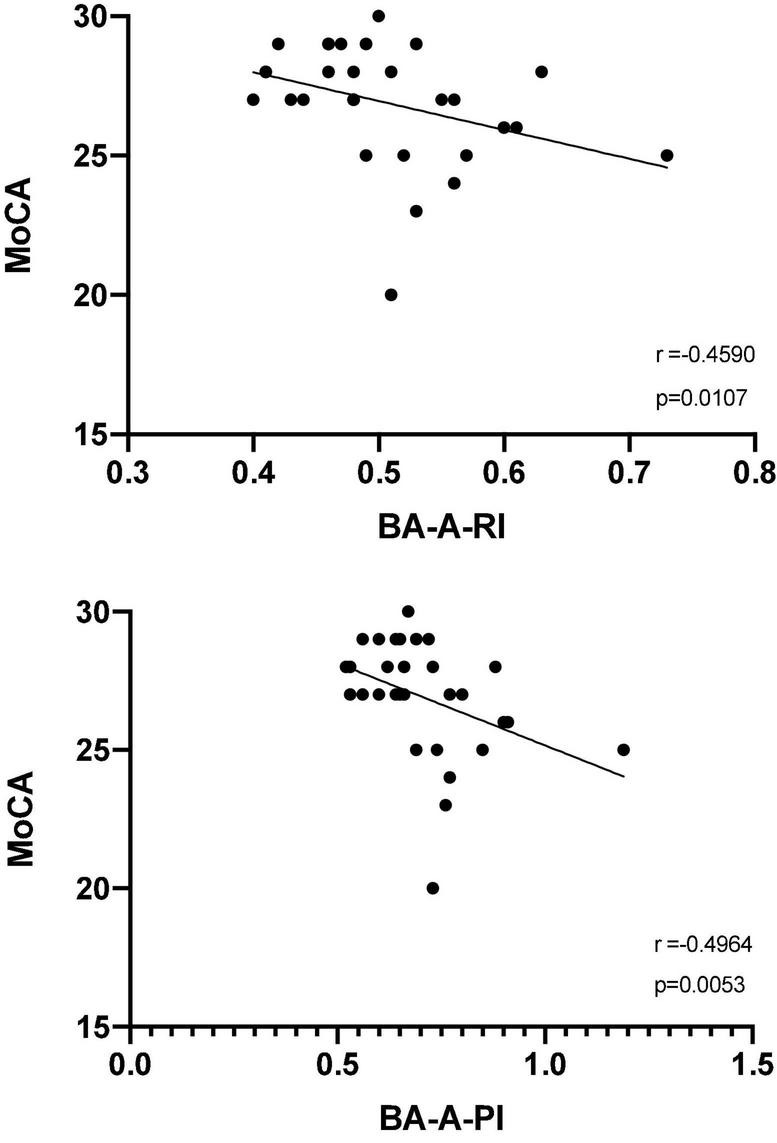
Spearman correlation analysis between Montreal cognitive assessment score (MoCA) and the resistivity index (top panel) and pulsatility index (bottom panel) from the basilar artery after the breath-holding test (BA-A-RI and BA-A-PI, respectively).

Although patients showed an overall trend toward higher values of CBF velocities from MCA bilaterally compared to controls, both at rest and after BHT, none of these comparisons reached a statistically significance; the same holds true for TCD measures of CBF velocity from BA at rest. Conversely, after BHT, both RI (0.53 vs. 0.48, *p* = 0.042) and PI (0.76 vs. 0.70, *p* = 0.023) were significantly higher in patients compared to controls (Table 2). The correlation analysis between clinical-psychocognitive and TCD data (Figure 1) revealed a statistically significant negative correlation between both indices from BA after BHT and MoCA score (RI: *r* = −0.459, *p* = 0.011; PI: *r* = −0.496, *p* = 0.005). Table 2 also shows the breath-holding index data (for BA and MCA, bilaterally), which did not significantly differ between CD patients and healthy controls.

## Discussion

### Summary of findings

This is the first report that systematically investigated both cerebral hemodynamics and vasoreactivity to TCD in a consecutive sample of treatment-naïve patients with CD with respect to age- and education-matched normal individuals. The main finding is a significant increase in both RI and PI from BA after BHT in patients, along with a negative correlation between both indices and cognition at MoCA. Overall, these results may suggest the presence of some subtle CVR changes in the posterior circulation of these newly diagnosed CD patients, the severity of which correlated with a worse cognitive performance.

The pathomechanisms explaining these results are likely to be quite complex, also because of the lack of any similar studies. A previous case report of a child with clinical and MRI findings of a right-sided stroke (Goodwin et al., 2004), reported significant asymmetry in the mean CBF, with increased right MCA velocity (127 cm/s, normal for age <114 cm/s; left MCA velocity 87 cm/s; difference 46%, normal <8%). Although there were no gastrointestinal symptoms, CD serology was positive and duodenal biopsy showed CD. At molecular level, the authors hypothesized that tTG can be involved in the maintenance of vascular endothelial integrity. Moreover, it has been demonstrated that anti-endomysial immunoglobulin A antibodies may react with cerebral vessels, thus hypothesizing an immune-mediated basis for CD-related stroke. Since CD may be a treatable cause of cerebrovascular diseases, anti-tTG antibodies need to be considered within the diagnostic work-up for strokes where no clear aitiology is found, even without the presence of typical gastrointestinal manifestations (Goodwin et al., 2004). Nevertheless, the patients here studied were adults without stroke/previous stroke or evidence of chronic cerebrovascular disease. Therefore, other mechanisms need to be invoked.

In this context, although little is known about CBF changes in CD, a previous single-photon emission computed tomography (SPECT) investigation in a restricted sample of CD subjects with cerebral calcifications and epilepsy demonstrated hypoperfusion of the occipital lobes, which extended to the temporo-parietal or frontal areas in most of them (Messa et al., 1997). Nevertheless, detailed data on CD and dietary status were not available and, as stated, all of these subjects exhibited a neurological involvement. A subsequent SPECT study in CD subjects without psychiatric or neurological involvement apart of depression or anxiety found evidence of hypoperfusion in the parietal and frontal regions of untreated participants, but not in those on GFD or in healthy controls (Addolorato et al., 2004). In particular, perfusion deficits were observed in the anterior and superior frontal cortex, which extended to the anterior cingulated cortex as well. Since similar abnormalities were described in psychiatric diseases (O’Connell, 2015), the hypoactivation of the prefrontal cortex (particularly in the left side), might be viewed as a depression marker (Grasby and Bench, 1997). Of note, this hypoactivation often recovered after an adequate therapy. The explanation for these observations is uncertain, although a molecular autoimmune etiology is likely (Tietge et al., 1997; Hadjivassiliou et al., 2003): since CD may be associated with autoimmune disorders (Farrell and Kelly, 2002), it cannot be excluded that cerebral perfusion changes might depend on immune complex–related or autoimmune endothelial inflammation, possibly involving antigliadin antibodies (Hadjivassiliou et al., 1996; Volta et al., 2002). However, the fact that our patients were neurologically unaffected (at least at the time of the study) may imply that these antigliadin antibodies are not always neurotoxic or that accessibility to CNS through blood/brain barrier breakdown is needed for such neurotoxicity to occur (Hadjivassiliou et al., 2002a). Abnormalities of brain perfusion in CD were also confirmed by other researchers (Usai et al., 2004). However, although this condition was similar to that earlier reported in other immune-mediated disorders, it did seem to be associated with autoimmunity and, at least in the frontal regions, might improve with GFD (Usai et al., 2004). Alternatively, the tissue deposition of antigliadin antibodies in CD may have interfered with enzymatic metabolic processes of the tracer within the neurons, thus causing the functional changes observed (Signore et al., 1997).

Regarding TCD indices, CBF velocities may be influenced by several factors, either physiological or pathological. An increase of this value is often reported in hyperdynamic flow, vasospasm, or vessel stenosis, while decreased value can reveal reduced CBF, hypotension, increased intracranial pressure, or brainstem death. Similarly, RI and PI can be affected by some components, such as vascular compliance, arterial pressure, and CO_2_ partial pressure changes. As such, PI provides information on downstream vascular resistance (Gosling and King, 1974; Naqvi et al., 2013), with occlusion or proximal stenosis lowering PI (likely secondary to downstream vasodilation of the arterioles), while constriction or distal occlusion increasing it (Nicoletto and Burkman, 2009). Modifications of RI reflect similar changes to those described for abnormal PI. Specifically, increased RI indicates enhanced downstream resistance, whereas arteriovenous malformation, hyperemia, rewarming following hypothermia, and vasospasm decrease it (White and Venkatesh, 2006; D’Andrea et al., 2016b).

Based on increased RI and PI from BA after BHT, we can hypothesize that a subclinical neurovascular involvement in CD might be associated not only with overt cerebrovascular events, but also with microstructural or functional changes likely due to an impaired vasoreactivity (Vinciguerra et al., 2020), which was more evident at the level of posterior brain circulation. This finding is in line with most electroencephalography findings in CD, which, although not disease-specific, exhibit bilateral or unilateral slow or spike waves, especially in the occipital areas (Pennisi et al., 2017a). Molecularly, the fact that occipital regions are commonly impaired in CD appears to be also confirmed by the finding of calcium deposition in these areas. The involvement of this region may depend on different reasons, including its susceptibility to some metabolic factors (e.g., hypoxia, hypoglycemia, etc.) and its structure, which is morphologically thinner than other cortices (Işikay et al., 2015). The predilection for the posterior circulation may also explain the fact that cerebellar dysfunction is by far the commonest neurological manifestation of CD. The question is whether these findings are cause or effect.

Clinical correlates of this finding may be found in the results of the cognitive test performed in this sample and the correlation between TCD measures and MoCA scores. Indeed, although a clear cognitive involvement was not detected, CD subjects scored significantly worse at MoCA with respect to controls. Furthermore, both TCD indices negatively correlated with MoCA, i.e., the higher are the indices the worse is cognition. In this scenario, it is known that some adults CD patients can report cognitive manifestations, mostly as a “brain fog,” which ameliorate after the adoption of the GFD, though they can appear again following casual gluten exposure (Lichtwark et al., 2014; Yelland, 2017). Difficulties in attention and concentration, lapses in word-retrieval and episodic memory, decreased psychic acuity, and disorientation or “confusion” episodes are also frequently complained (Lurie et al., 2008). In severe patients, fortunately today more and more rare, an overt dementing disorder may manifest (Collin et al., 1991; Hu et al., 2006; Lurie et al., 2008; Casella et al., 2012). In this scenario, transcranial magnetic stimulation (TMS), an electrophysiological technique that may non-invasively assess and monitor different neurological and neuropsychiatric disorders (Cantone et al., 2020, 2021; Fisicaro et al., 2020; Di Lazzaro et al., 2021), even those without clear clinical manifestations (Lanza et al., 2020a,b), has been recently applied to CD patients, both at diagnosis (Pennisi et al., 2014; Lanza et al., 2021) and after a short- or long-term

GFD (Bella et al., 2015; Pennisi et al., 2017b). Overall, these studies suggest a global pattern of intracortical and intercortical “hyperexcitable celiac brain,” which partially recovers with a long lasting GFD (Lanza et al., 2018, 2021; Fisicaro et al., 2021). Translationally, this hyperexcitability to TMS may represent the neurophysiological correlate of the hyperdynamic circulation to TCD, although, at this stage, an association only and not a causal or reciprocal effect can be postulated.

Finally, the psychiatric comorbidity, namely anxiety and depression is commonly reported in CD (Obrenovich, 2018; Slim et al., 2018). In this sample, features of depression were higher compared to controls, though the mean score of HDRS was compatible with mild symptoms. More in general, mood changes may impair the quality of life of subjects with CD and significantly affect the compliance to GFD (Muhammad et al., 2019). Therefore, detection and monitoring of depression are of pivotal importance to refer for adequate pharmacological treatment and/or psychotherapy. Recent molecular findings have also proposed the role of some soluble inflammatory factors from the gut damaged mucosa across the gut–epithelial barrier and the blood–brain barrier as relevant components for both functional and structural changes at the brain level. Namely, it has been widely documented that neuroinflammatory phenomena negatively impact both emotional behavior and cognition (Crupi et al., 2010; Günther et al., 2021), thus adding further support to the relationship between CD, neuropsychiatry, and neuroinflammation. This evidence underlines the need for an early diagnosis and full adherence to the GFD to limit, or even prevent, the neuropsychiatric and neurological involvement in CD and its complications (Hadjivassiliou et al., 2019).

### Limitations

First, a main caveat of this investigation is the relatively small sample size, though CD subjects were carefully selected for histopathological features and serological-clinical data; moreover, all of them were *de novo*, drug-free, and matched for sex, age, education, and anthropometric features with normal controls. In order to avoid the interobserver variability, all measurement were carried out by the same sonographer and with the same instrumentation.

Another caveat is that CT has a general lower sensitivity and specificity than MRI, although it can identify intracranial calcifications more reliably than MRI. The same holds true for EEG (not performed) and an exhaustive battery of neuropsychological tests, which was restricted to MoCA, which is however a comprehensive test of global cognitive functioning.

Third, given the cross-sectional study design, it needs to be acknowledged that, at this stage, a direct correlation between CD and TCD cannot be drawn, but only an association between gluten exposure and specific hemodynamic indices. Follow-up studies in gluten-restricted patients will further clarify their clinical and pathophysiological impact.

Finally, it is known that anemia with low hematocrit value may influence TCD parameters (Brass et al., 1988; Ameriso et al., 1990). However, as per the above-stated inclusion/exclusion criteria, anemia was classified as mild (hemoglobin level ranging from 10.0 g/dL to the lower limit of normal) in all the affected patients, with a hematocrit value no lower than 32% in all female subjects and of 35% in the only male subject affected. Therefore, although a potential effect of anemia in this sample cannot be entirely excluded, the presence of mild anemia, without markedly low hematocrit value, may have not significantly affected the observed results, as also previously demonstrated (Aliefendioglu et al., 2007). Also, it seems unlikely that anemia would have resulted in preferential abnormalities in the posterior circulation, as opposed to more global changes.

Lastly, TCD parameters were not measured from the anterior cerebral artery and the posterior cerebral artery; nevertheless, BA and MCA irrorate most of the posterior and anterior brain areas, respectively, and are standardized for TCD examination, thus providing a satisfactory and reliable evaluation of cerebral resistance and velocities.

## Conclusion

Transcranial Doppler sonography in CD may disclose some subtle CVR changes in the posterior circulation of these neurologically asymptomatic patients, thus possibly suggesting its role as an additional tool to non-invasively probe the cerebrovascular correlates of CD. Follow-up investigations, which correlate neuroimaging and clinical results, both at the time of diagnosis and after the gluten restriction, should be encouraged to confirm this finding and to gain further insights on the fascinating connections between gut and brain.

## Data availability statement

The original contributions presented in this study are included in the article/supplementary material, further inquiries can be directed to the corresponding author.

## Ethics statement

The studies involving human participants were reviewed and approved by The Ethics Committee of “G. Rodolico-San Marco” Policlinico University Hospital of Catania (Italy) (Prot. n. 103/694). The patients/participants provided their written informed consent to participate in this study. Written informed consent was obtained from the individuals for the publication of any potentially identifiable images or data included in this article.

## Author contributions

FF and MP: conceptualization. RB: methodology, investigation, and project administration. GL, CD’A, and MP: validation. MC: formal analysis and data curation. CD’A: resources. FF and GL: writing—original draft preparation. GP and MH: writing—review and editing. MH: visualization. GP: supervision. All authors have read and agreed to the published version of the manuscript.
